# In-vitro study of the wear behavior of telescopic crowns made of CoCr, zirconia or PEEK

**DOI:** 10.1007/s10856-025-06891-6

**Published:** 2025-05-14

**Authors:** PC. Pott, N. v. Maltzahn, C. Brachmann, M. Stiesch, P. Kohorst

**Affiliations:** 1 Department of Prosthetic Dentistry and Biomedical Materials Research, Hannover Medical School, Carl-Neuberg-Str. 1, 30625 Hannover, Germany; 2 Privatpraxis für Zahnmedizin, Prof. Dr. Philipp Kohorst, Lilienthaler Heerstr. 261, Bremen, Germany

## Abstract

Purpose: Double-crown-retained dentures (DCRD) are an established treatment in dentistry. In some cases, the retention between primary crown (PC) and secondary crown (SC) loosens due to wear during daily handling of the denture. The aim of the present study was to examine modern materials for PCs and SCs with regard to their wear behavior. Materials and Methods: Cyclic pull-off tests were conducted on each 40 specimens in five test groups. PCs made of CoCr, PEEK and ZrO_2_ were cemented onto a prepared resin tooth. SCs were made of the same materials and combined to the following PC-SC combinations: CoCr-CoCr, CoCr-PEEK, PEEK-PEEK, ZrO_2_-PEEK, ZrO_2_-ZrO_2_. The wear areas on the contact surfaces between the PCs and the SCs were examined on SEM images. Results: Wear marks in form of scratches, chipping, ductile deformation or combinations of these forms were observed on the PCs and SCs in the various material combinations. The greatest wear on PCs was observed in the PEEK-PEEK group, while the greatest wear on SCs was observed in the ZrO_2_-ZrO_2_ group. The least wear was observed on SCs made of PEEK in combination with PCs made of ZrO_2_. Conclusion: When using modern materials for DCRDs, the combination of different materials for PCs and SCs must be taken into account. The data found in this study show that only minor wear occurs at SCs made of PEEK, especially in combination with PCs made of ZrO_2_. Therefore, this combination seems particularly suitable for increasing the longevity of DCRDs.

**Graphical Abstract:**

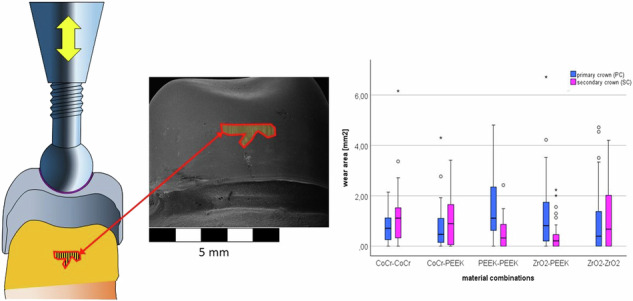

## Introduction

Double-crown retained removable dentures (DCRD) are an established treatment method for patients with severely reduced residual dentition, usually with very good and long-term treatment results. In principle, DCRDs are characterized by high positional stability, good hygienic properties, good esthetics and excellent expandability in case of further tooth-loss. In their work published in 2023, *Kopzon* and *Raedel* were able to show that even after 27 years of clinical use, DCRDs still remain a clinically successful treatment method [1]. *Widbom* et al. were able to show that the wear of the prosthesis itself becomes a problem for the wearers over time [2]. *Zafiropoulos* et al. retrospectively analyzed the long-term clinical success of tooth-supported DCRDs and found a survival rate of 99.3%. However, the success rate was significantly lower at 77%. The risk of mechanical complications was significantly higher for cantilever prostheses than for prostheses used to treat interdental gaps [3]. *Schwindling* et al. evaluated DCRDs after seven years of clinical use. They observed mainly simple complications, such as decemented primary crowns, denture fractures or fractures of the veneering material. The resulting survival rate was 93%. They also compared telescopic crowns with conical crowns and found a higher success rate for telescopic crowns [4].

Despite the fact that DCRDs are a well-established therapeutic tool and that complications can be easily managed in most cases, even after a long period of use a remaining problem with DCRDs is the loss of retention between the primary crown (PC) and secondary crown (SC), which occurs due to material wear when the dentures are inserted and removed in daily routine. *Luft* et al. confirmed this retention loss as one of the major problems with DCRDs [5]. The wear behavior of double crowns depends on various parameters. The geometry of the double crowns themselves plays a role, as does the construction of the denture [4]. The number of double crowns integrated into a DCRD is also relevant [6]. However, the combination of the materials used and how they are manufactured must be considered in a special way.

Cast gold alloys are still current standard for tooth retained double crown systems. They are becoming increasingly expensive and are being replaced more and more by different non-precious metal alternatives, not least due to the improved quality resulting from digital processing techniques. In addition to metal alloys and ceramic materials, modern computer aided design and manufacturing (CAD/CAM) techniques allow the processing of high-performance polymers such as polyetheretherketone (PEEK) or polyetherketoneketone (PEKK). *Luft* et al. conclude, that modern double crown systems manufactured by CAD/CAM are similar to the actual gold standard using cast gold alloys [5]. *Emera* and *Askar* identified poly-ether-ketone-ketone (PEKK) as a promising material for SCs combined to PCs made of zirconia after 6 month of clinical use [7]. *Hamid* et al. compared different material combinations for PCs and SCs after cyclic loading for 1 × 10^3^, 5 × 10^3^ and 10 × 10^3^ cycles. They found significantly less wear for PEEK-PEEK, than for combinations of PEEK with zirconia or cobalt-chrome alloy [8]. Unfortunately, in total there is still a lack of data about wear behavior of double crown systems in the literature. Above all, data is still lacking on where to expect wear and tear in which material combinations, which ultimately has a significant influence on the aftercare measures to be taken.

Aim of the presented study was to evaluate the wear of telescopic crowns made of different material combinations for PC and SC after artificial aging under simulated intraoral conditions.

## Materials and methods

**Fig. 1:**
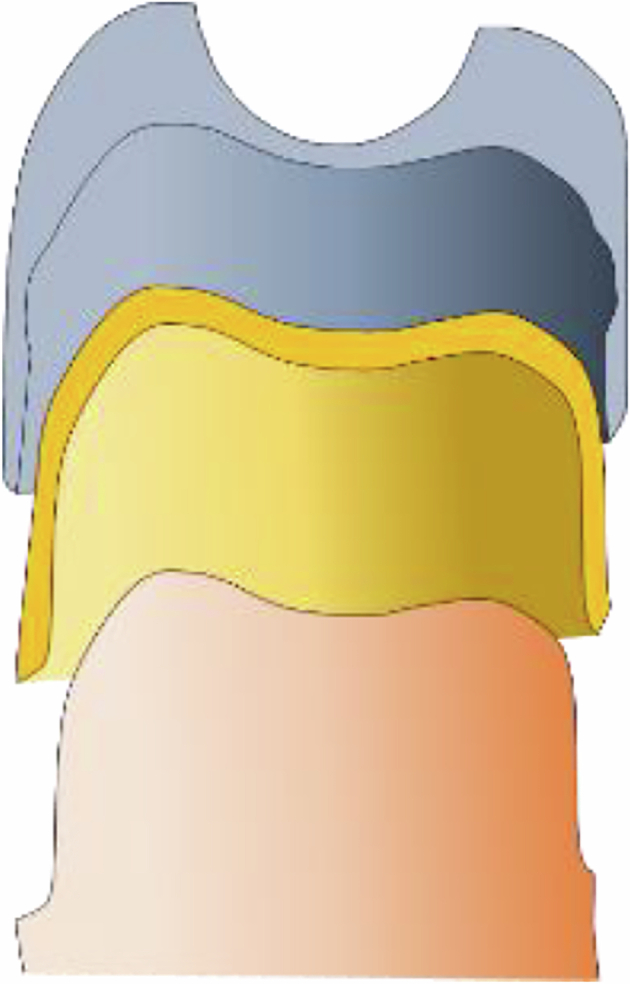
Schematic drawing of a prepared tooth (orange), the primary crown (yellow) and secondary crown (grey)

The presented study is an in vitro study. First, an upper premolar was prepared for a telescopic crown on a model according to the preparation guidelines of the Hannover Medical School with a circumferent champfer preparation and a preparation angle of 3 degree to the tooth axis (Fig. 1). A duplicate mold (remaSIL Typ 2 108-700-00, DENTAURUM, Ispringen, Deutschland) was created from this preparation, which was used to fabricate 200 identical test models from abrasion-resistant model resin (Alpha Die, Schütz Dental GmbH, Rosbach von der Höhe, Germany). The bases of all models were trimmed flat at a 90° angle to the tooth axis.

In a dental laboratory (Zahntechnik Xental Celle GmbH, Nienhagen, Germany), the prepared tooth of the original model was digitized. Subsequently, both the primary crown and the secondary crown were designed digitally. On the basis of these data sets, 80 PCs were then made of a CoCr alloy (CoCr BioStar, Siladent Dr. Böhme & Schöps GmbH, Goslar, Germany), 80 PCs of zirconia (ZrO_2_) (High strength zirconia, Dental Direkt GmbH, Spenge, Germany) and 40 PCs of Polyetheretherketone (PEEK) (JUROVA Dental Disc, JUROVA Ltd, Lancs, Great Britain). In addition, each 40 SCs were made of CoCr alloy and zirconia. Further 120 SCs were made of PEEK. Accordingly, the following material combinations were used in five test groups of 40 specimens each: CoCr-CoCr, CoCr-PEEK, PEEK-PEEK, ZrO_2_-PEEK, ZrO_2_-ZrO_2_.

To prepare the individual test specimens, the luminal surfaces of the PCs were sandblasted with 110 µm aluminum oxide prior to cementation on the prepared tooth, degreased with alcohol and dried with oil-free compressed air. All PCs were cemented to the prepared tooth with dual-curing composite cement Panavia 2.0 (kuraray Noritake, Tokyo, Japan) according to the respective material-specific manufacturer’s instructions.

**Fig. 2:**
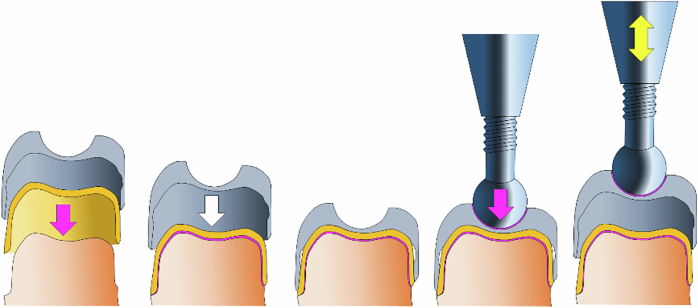
Schematic drawing of the experimental setup: (from left to right) the PC gets luted adhesively onto the prepared tooth (pink arrow), the SC is placed onto the primary crown without luting (white arrow), complex of PC and SC on the tooth, ball-head screw gets luted onto the SC, the SC can be lifted from and put back onto the PC (yellow double-headed arrow)

The lumen of the SCs was designed in such a way that it has a band-shaped friction surface of 1.5 mm in width at a small distance from the crown margin of the PC above the ending of the champfer. When multiplied by the inner circumference of the SCs in the area of the friction surface of 25.7 mm, the result is a contact surface between the PC and SC in the end position of 38.5 mm^2^. In combination with the 1 mm lifting height during the cyclic loading, the possible contact area on the PC is 64.2 mm². The SCs are equipped on their occlusal surface with a mold corresponding to a ball of 4 mm diameter. For the pull-off tests a testing machine (DYNA-MESS Prüfsysteme GmbH, Aachen, Germany) was equipped with a ball head screw, also with a diameter of 4 mm. The test specimen with cemented PC and SC placed on it was positioned under the ball head of this screw in such a way that the ball head was precisely positioned in the corresponding mold of the SC. The ball head was then also adhesively bonded (Panavia 2.0, kuraray Noritake, Tokyo, Japan) in the mold without tension. All samples were stored on a silicone base during the artificial ageing process. This helped to minimize positional inaccuracies. The cyclic tensile tests were carried out under water at 36°C to simulate the conditions in the oral cavity. For each test specimen, 10 x 10^3^ load cycles were performed at a frequency of 1 Hz (Fig. 2). Assuming that a telescopic prosthesis should be inserted and removed four times a day, this number of load cycles corresponds to a simulated aging of 6.8 years.

**Fig. 3:**
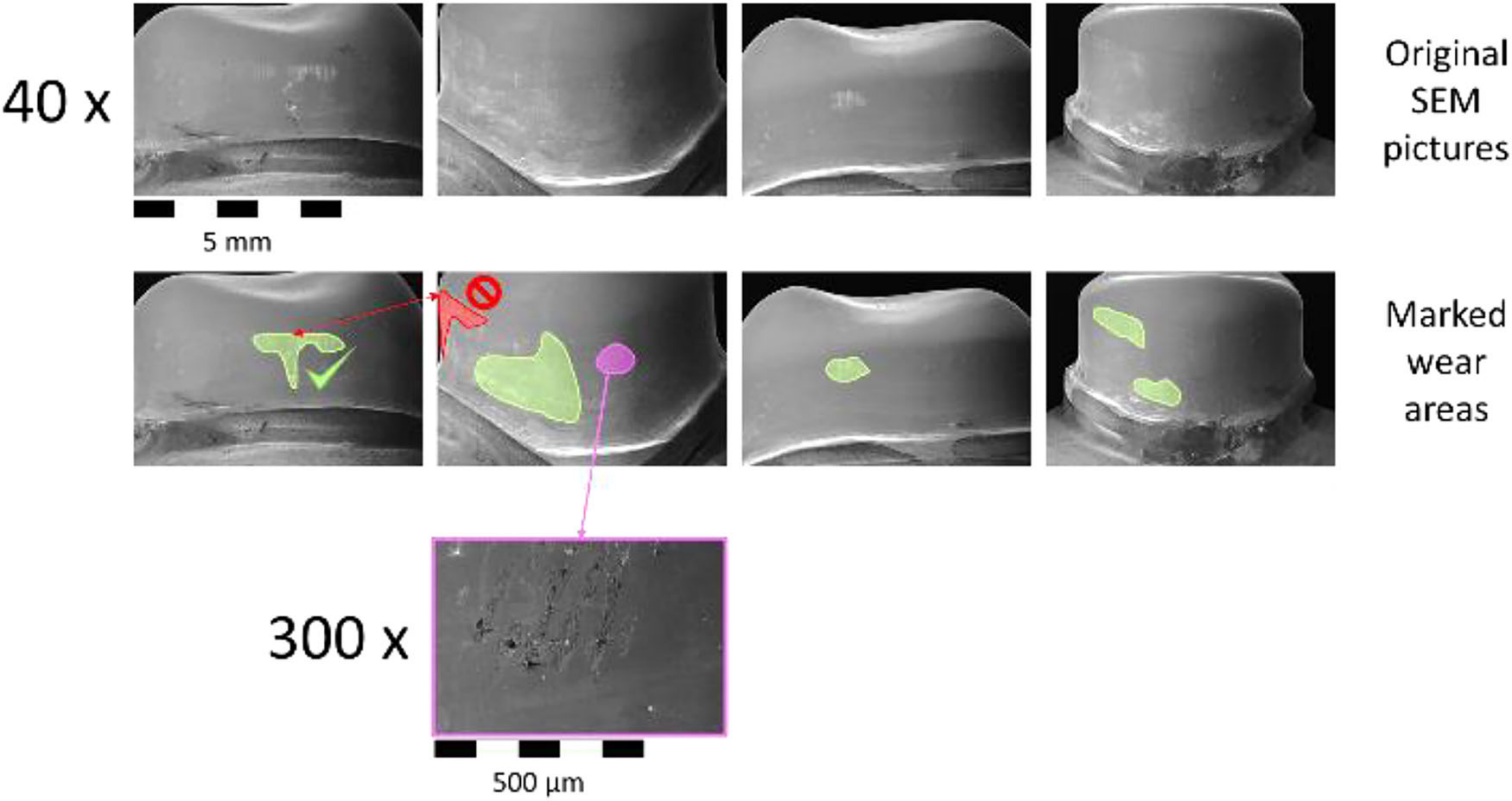
Identification of the wear areas in the SEM pictures of an examle primary crown. All of the SEM-Images were taken using the following parameters: 40x Images (EHT = 12.00 kV, Detector = SE1, Vacuum 1.16e-004 Pa. 8 pA, WD 46.0 mm, Width 7.5 mm), 300 x (EHT = 3.00 kV, Detector = SE1, Vacuum 4.68e-005 Pa. 300 pA, WD 23.0 mm, Width 1.0 mm)

The examination of the abrasions was carried out using a scanning electron microscope. First, SEM images were taken of three randomly selected PCs and SCs made of the different materials at 40x and 300x magnification from the vestibular, mesial, palatal and distal sides before loading to document the initial situation. After the loading test, the PCs and SCs of the test specimens were again examined under the SEM, initially at 40x magnification. If necessary, higher resolutions were used to differentiate between the types of wear. SEM-Images were taken using the following aquisition parameters: 40x Images (EHT = 12.00 kV, Detector = SE1, Vacuum 1.16e-004 Pa. 8 pA, WD 46.0 mm, Width 7.5 mm), 300 x (EHT = 3.00 kV, Detector = SE1, Vacuum 4.68e-005 Pa. 300 pA, WD 23.0 mm, Width 1.0 mm). The size of the wear area detected was defined as a measure of the wear intensity of the respective material combinations. For this purpose, the wear areas were identified and added up in the four SEM images per specimen. To avoid counting the same area twice, it was determined beforehand which measuring areas on which SEM images would be evaluated, thereby the wear areas were measured in the picture with the lowest distortion (Fig. 3).

This results in the difficulty of measuring a three-dimensionally curved surface on a two-dimensional representation of this surface. However, since the aim of this study is not to compare the absolute wear area, but to compare the wear between different material combinations, this lack of clarity does not play a significant role in the interpretation of the data.

For the statistical analysis, the homogeneity of variance was verified using Levene’s analysis, and the normal distribution was confirmed using the Kolmogorov-Smirnov test. The statistical comparison of the groups was carried out accordingly using ANOVA and Tukey HSD (IBM SPSS Statistics 29.0.1.0, IBM, USA, Armonk, New York).

## Results

**Fig. 4:**
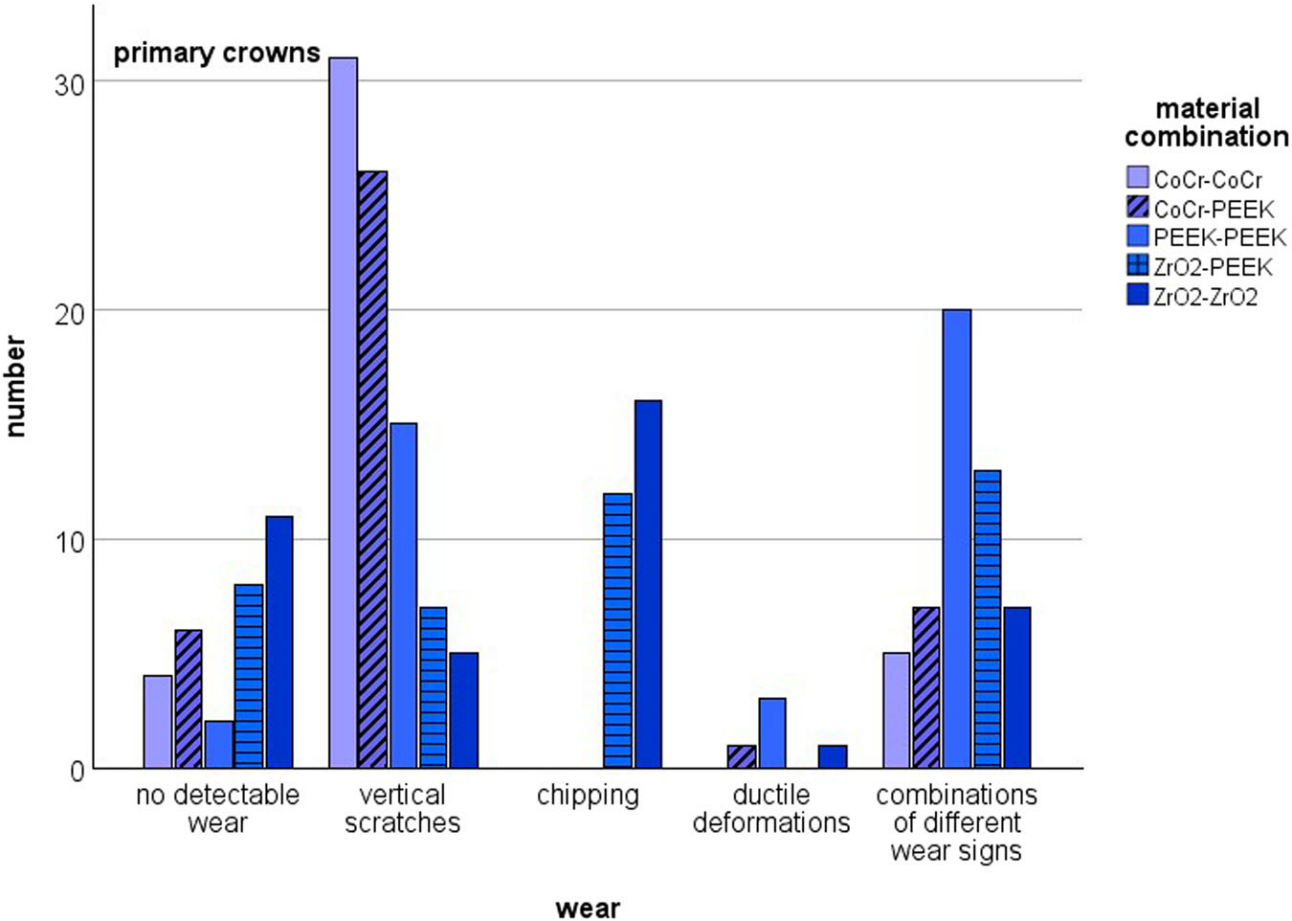
Distribution of the types of wear in the PCs

**Fig. 5:**
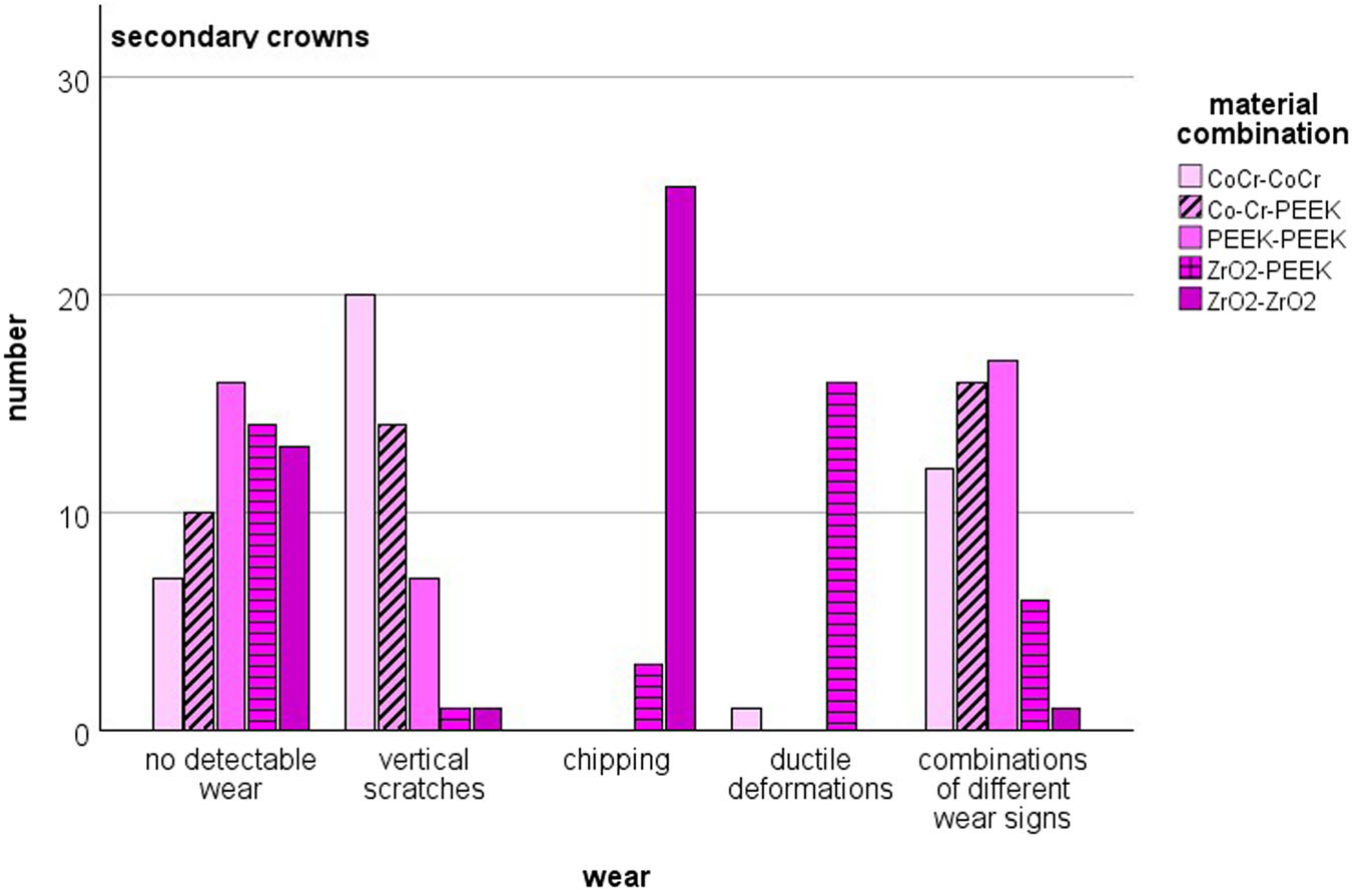
Distribution of the types of wear in the SCs

SEM examination of the PCs and SCs revealed various forms of wear. Some showed no wear at all, while scratches, chipping, ductile deformation or combinations of the above forms of wear were observed. The bar charts in Figs. 4 and 5 show the distribution of wear on the material pairings for PCs and SCs (Fig. 4, Fig. 5). Signs of wear occurred in all material combinations, both on the PCs and on the SCs. In some cases, no signs of wear were detected. In many cases, combinations of vertical scratches, chipping or ductile deformation of the materials were found. When comparing PCs to SCs, scratches were more frequently detected on PCs, while chipping was more common on SCs, especially when both PC and SC were made of zirconia.

**Fig. 6:**
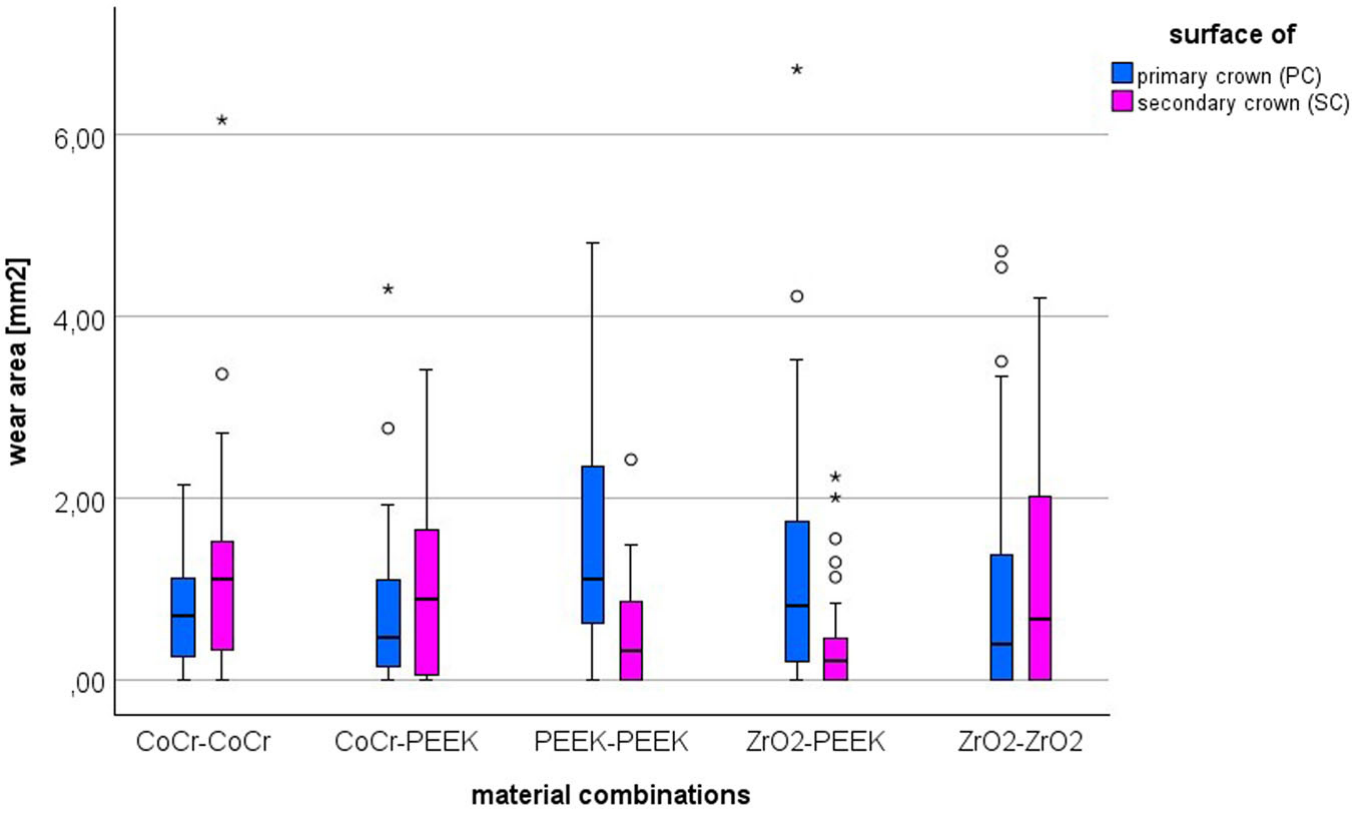
Boxplots of the sum of the wear marks areas on the primary crowns and secondary crowns in the 5 material pairings in SEM pictures

For the abrasion analysis of the PCs and the SCs, the areas with vertical lines or other visible wear marks were identified on the SEM images for each sample. These vertical lines are wear marks caused by the lifting movements between the PC and SC. The corresponding correlating areas were measured and added for all four images. The resulting data are shown in the boxplot diagram (Fig. 6).

First, the wear area of the primary crowns was assessed. The Kolmogorov-Smirnov test confirmed the normal distribution of the data (*p* < 0.001), and the Levene analysis confirmed the homogeneity of variance (*p* < 0.001). Using ANOVA, it was possible to show that the test groups differ significantly (*p* = 0.012). The corresponding individual comparisons using the Tukey-HSD test showed that the wear surface of the PEEK-PEEK pairing was significantly larger compared to CoCr-CoCr (*p* = 0.026) and CoCr-PEEK (*p* = 0.025). All other individual comparisons remained without statistical significance. The secondary crowns were evaluated afterwards. Normal distribution and homogeneity of variance were confirmed again (*p* < 0.001). The ANOVA also showed significant differences between the groups for the SCs (*p* < 0.001). The corresponding individual comparisons were again carried out using Tukey-HSD. It was found that significantly more wear was visible in CoCr-CoCr than in PEEK-PEEK (*p* = 0.005) and ZrO_2_-PEEK (*p* = 0.002). CoCr-PEEK also showed significantly more wear marks in the SC than ZrO_2_-PEEK (*p* = 0.039). The most wear marks were found in the SC of the ZrO_2_-ZrO_2_ pairing. These were significantly higher compared to PEEK-PEEK (*p* = 0.023) and ZrO_2_-PEEK (*p* = 0.008). All other individual comparisons remained without statistical significance. When comparing the wear marks between the PC and SC within the individual material combinations, it was shown that SCs made of PEEK showed significantly less wear when the PCs were also made of PEEK or ZrO_2_.

## Discussion

In the study presented, a few limiting factors had to be dealt with. When adjusting DCRDs, precise setting of the required withdrawal forces between PCs and SCs is generally very important. This is done manually in the dental laboratory. Therefore, minimal differences in the withdrawal forces of several double crowns are unavoidable, even if the geometry of the individual PCs and SCs is identical. In this study, this problem was counteracted by selecting a very large group size of 40 specimens per group. In addition, all PCs and SCs were manufactured using the CAD/CAM method based on a single data set. According to *Arnold* et al., CAD/CAM fabrication can provide a better fit of telescopic crowns than manual fabrication [9].

Another methodological limitation of this study is the analysis of three-dimensional surfaces using two-dimensional SEM data sets. The evaluation of absolute areas is not possible due to the curvature of the PCs and the associated distortion in the two-dimensional representation. However, in the present study, both the PCs and the SCs had identical shapes and the SEM images were acquired and evaluated in an identical manner. The corresponding distortion was therefore present to the same extent in all samples. It can therefore be neglected as an influencing factor when comparing the abrasion data between the individual material combinations.

In general, friction loss by wear still remains a problem in DCRDs [10]. In this study, a standardized experimental design was chosen under controlled laboratory conditions to simulate the use of DCRDs. In this process, the wear of the contact surfaces between the PC and SC is simulated exclusively in the correct insertion axis between the PC and SC. In clinical practice, patients often wiggle the prostheses to loosen the retention and to loosen the prostheses when more than one telescopic crown is combined in a DCRD. The tilting movements can lead to additional wear on the telescopes. Patients only learn how to use the new restoration properly after an initial, patient-specific familiarization phase [11]. It must be assumed that there may be increased abrasion between the PCs and SCs during this familiarization phase, since the correct insertion axes have not yet been properly established. *Bayer* et al. were able to show that the retention forces between the primary and secondary crowns remain stable for the first 18 months due to wear. After that, they decrease, but are still sufficient to anchor the prosthesis [12]. This effect cannot be depicted in the study design chosen for this study.

The literature contains various data on the simulated duration of ageing that can be achieved with 10 × 10^3^ cycles in lift-off tests. Priester et al. state that 10 × 10^3^ cycles correspond to an aging of 10 years [13]. In the present study, it is assumed that 10 × 10³ cycles correspond to only 6.8 years of aging. This is based on the assumption that denture wearers insert their dentures in the morning, remove and rinse them after each meal, and remove them again at night. Based on three main meals a day, this results in a total of four insertion and removal processes per day (10 × 10^3^ / 4 = 2,5 × 10^3^ / 365 = ~6.8).

The materials used for PCs and SCs are usually a combination of metals or ceramics. Established combinations include gold-gold, CoCr-CoCr or ZrO_2_-gold. The high cost of precious metals and the complex processing of non-precious metals in particular make it necessary to establish new materials for PCs and SCs. *Liu* et al. have confirmed in their extensive work that PEEK has a wide range of applications in the field of removable dentures [14]. *Priester* et al. analyzed comparable material combinations for PCs and SCs. In contrast to the presented data, their analyses based on an upper first molar. They found the highest retention forces for the combination PEEK-PEEK [13]. In 2024 *Emera and Askar* conclude, that poly-ether-ketone-ketone (PEKK) is a promising material for SCs combined to PCs made of zirconia [7]. Although the study presented dealt with wear and not with the retention forces of double-crown systems, comparability can be found here. The results of the presented study show that the material combination plays a relevant role in the wear between PCs and SCs. When the PC and SC were both made of CoCr, the wear between the PC and SC was almost the same. This was also the case when the PC and SC were made of zirconia. In the other groups, PCs showed much more wear than the SCs. This is in line with *Güven* et al. who also found wear mostly in primary crowns [15]. When PEEK was used as the material for the SCs, significantly more wear was observed on the SC when combined with CoCr. This can be explained by the greater material hardness of the metal alloy. *Luft* et al. have made similar observations [5]. When combined with ZrO_2_, the PEEK SC showed significantly less wear. This is due to the fact that the surface of the zirconia PC is significantly smoother than the metal. In general, less wear was observed in PEEK-containing heterogeneous material combinations. This is also consistent with the results of *Luft* et al. [5] and *Turp* et al. [16]. It was interesting to note that the greatest signs of wear were observed on the primary crown and significantly less wear on the secondary crown in the PEEK-PEEK combination. In the opinion of the authors, this is because the secondary crowns deformed minimally during the tests, which is why no signs of wear were often visible in the SCs in this group. Such deformation could also be the cause of the even lower wear of the PEEK SCs in combination with the zirconia PCs.

In addition to the above-mentioned methodological limitations of the study, the following aspects must also be considered when interpreting the data. In addition to the materials used, the design of the double crowns also plays a role with regard to the respective adhesion mechanism and, accordingly, the wear behavior. *Schimmel* et al. conclude that the design of precious alloy telescopic crowns cannot be transferred to milled materials combinations [17]. *Bayer* et al. were able to show that there were differences in the pull-off forces between cast and electroplated SCs, which has a direct influence on wear. However, they conclude that both design principles can be used in clinical practice [6]. In the present study, the pull-off tests were only carried out on one telescopic crown at a time. So there is a clear path of insertion and pull-off for the crown. Irrespective of the materials used, the retention force increases, if two or more telescopes are combined in one denture [13]. The investigation of such effects must be the subject of future research.

## Conclusion

Despite the limitations of this study, the following can be concluded: When constructing telescope-supported dentures, the materials for PC and SC must be chosen very carefully. Materials of similar hardness for PC and SC lead to good results and should be used preferentially. PEEK as a material for SCs is particularly interesting in combination with ZrO_2_ PCs, as only minor wear of the SCs has been observed. It is therefore to be expected that the longevity of the DCRDs would benefit in this case. The material thickness of the SCs should then be selected to avoid deformation of the SCs.

## Data Availability

The datasets used and/or analysed during the current study are available from the corresponding author on reasonable request.

## Abbrevaiations

ANOVA: Analysis of Normal Variation
CAD/CAM: Computer aided design / Computer aided manufacturing
CoCr: Cobalt-Crome alloy
DCRD: double-crown retained removable denture
PEEK: Poly-Ether-Ether-Ketone
PEKK: Poly-Ether-Ketone-Ketone
PC: primary coping
SC: secondary coping
SEM: Scanning Electron Microscopy
ZrO_2_: zirconia
